# Application of ABR in pathogenic neurovascular compression of the 8th cranial nerve in vestibular paroxysmia

**DOI:** 10.1007/s00701-022-05157-2

**Published:** 2022-03-05

**Authors:** Huiying Sun, Xu Tian, Yang Zhao, Hong Jiang, Zhiqiang Gao, Haiyan Wu

**Affiliations:** Department of Otorhinolaryngology, Perking Union Medical College Hospital, Chinese Academy of Medical Sciences Peking Union Medical College, No.1 Shuaifuyuan,Wangfujing, Dongcheng District, 100730 Beijing, China

**Keywords:** Vestibular paroxysmia, Neurovascular compression, Auditory brainstem response, Magnetic resonance imaging

## Abstract

**Purpose:**

To investigate the clinical value of electrophysiological tests in indicating pathogenic vascular contact of the 8th nerve in definite vestibular paroxysmia (VP) cases to provide a reference for decompression surgery.

**Methods:**

We retrospectively analyzed patients who had vertigo, unilateral tinnitus, or hearing loss and exhibited vascular contact of the 8th cranial nerve by MRI. Participants were classified into the VP or non-VP group according to the criteria of the Bárány Society in 2016. The demographic characteristics and audiological and electrophysiological test results of the two groups were compared. Receiver operating characteristic (ROC) curves were calculated for ABR to determine the best parameters and cutoff values to predict the existence of pathological neurovascular contact in VP.

**Results:**

Thirteen patients in the VP group and 66 patients in the non-VP group were included. VP patients had longer interpeak latency (IPL) I–III and wave III latency compared to non-VP patients (*p* < 0.001; *p* < 0.001). According to the ROC analyses, IPL I–III and wave III latency were the best indicators for the diagnosis of VP. The optimal cutoff for IPL I–III was 2.3 ms (sensitivity 84.6%, specificity 95.5%), and that for wave III latency was 4.0 ms (sensitivity 92.3%, specificity 77.3%). There were no differences in the PTA, caloric test, o-VEMP, or c-VEMP results between the two groups.

**Conclusion:**

Prolonged IPL I–III and the wave III latency of ABR strongly suggested that vascular contact of the 8th cranial nerve was pathological, which may provide some references for microvascular decompression surgery of VP.

**Supplementary Information:**

The online version contains supplementary material available at 10.1007/s00701-022-05157-2.

## Introduction

Neurovascular cross-compression (NVCC) in the cerebello-pontine angle (CPA) or internal acoustical canal (IAC) may cause vertigo, tinnitus, or hearing loss [13, 14, 25]. Vestibular paroxysmia (VP), previously termed “disabling positional vertigo,” is a certain kind of NVCC of the 8th cranial nerve that results in spinning or non-spinning dizziness, with or without ear symptoms such as tinnitus, sensorineural hearing loss, and acoustic hypersensitivity [4]. The diagnostic criteria for VP were defined by the Classification Committee of the Bárány Society in 2016 [27]. The prevalence of VP is relatively low, at less than 1 in 2000 people [27], and VP is frequently reported from 40 to 50 years of age [3, 28]. A low dose of carbamazepine or oxcarbazepine is the preferred treatment for VP. However, some patients do not respond well to pharmacotherapy or cannot tolerate the side effects of these drugs [32]. For these patients, microvascular decompression surgery is a choice that was introduced for hemifacial spasms, trigeminal neuralgia, and refractory vertigo [11, 14, 21]. But this surgery may lead to serious postoperative complications such as hearing loss, facial palsy, and cerebrospinal fluid leakage [22, 31], and the surgical indications remain uncertain in VP [24]. To avoid unnecessary surgeries, adequate preoperative assessments providing evidence for pathogenic neurovascular contact are essential prerequisites.

Over 95% of VP patients show neurovascular contact on magnetic resonance imaging (MRI) with relatively high sensitivity (nearly 100%) and low specificity (65%) [1, 26]. It is difficult to determine if neurovascular contact is problematic or if it leads to the onset of VP because some patients with contacts on MRI are asymptomatic. Previous studies have reported some changes in electrophysiological tests, such as auditory brainstem response (ABR), caloric tests, and vestibular evoked myogenic potentials (VEMPs) [5, 9, 16], but their clinical value in determining whether the neurovascular contact shown in radiology is pathological remains uncertain and controversial. Thus, this paper aimed to investigate the outcomes of these electrophysiological tests and to assess the possibility of utilizing these tests as an indicator for neurovascular conflict in VP patients with neurovascular contact demonstrated on MRI, providing some objective references for microvascular decompression surgery and aiding in decision-making for potential candidates. This study was exempted from the Institutional Review Board review by the Medical Ethics Committee of Peking Union Medical College Hospital (S-K1695).

## Materials and methods

### Participants

The medical charts of patients diagnosed with vertigo, unilateral subjective tinnitus, or unilateral hearing loss with vascular contact of the 8th cranial nerve on the affected side confirmed by MRI and presented at the Department of Otorhinolaryngology in Peking Union Medical College Hospital from September 2019 to November 2020 were retrospectively analyzed. The exclusion criteria were otitis media, tumor of the ear or cerebellopontine angle, and head or ear trauma. Patients who met the criteria for definite VP by the Bárány Society in 2016 [27] were classified into the VP group, and the others were classified into the non-VP group. The criteria for definite VP were as follows: ① at least ten attacks of spontaneous spinning or nonspinning vertigo; ② duration less than 1 min; ③ stereotyped phenomenology in a particular patient; ④ response to treatment with carbamazepine/oxcarbazepine; and ⑤ not better explained by another diagnosis. The affected side of all the patients was verified according to the comprehensive considerations of symptoms, hearing level, and results of electrophysiological testing.

The demographic characteristics of all the patients were collected. Tinnitus was recorded as typewriter (staccato, not pulse synchronous), pulsatile, and persistent (low- or high-frequency) types. Vertigo was divided into acute, episodic, and chronic vestibular syndromes [2]. The hearing level was assessed according to the mean value of the thresholds of 0.5, 1, 2, and 4 kHz by pure tone audiometry (PTA). All participants underwent vestibular electrophysiological evaluations, including ABR, caloric test, ocular vestibular evoked myogenic potential (o-VEMP), and cervical vestibular evoked myogenic potential (c-VEMP), in a sound-proof booth. The ABR results were further analyzed according to the criteria by Møller [22], including latencies of waves I, III, and V, interpeak latency (IPL) I–III and IPL III–V, and IPL I–III difference and IPL III–V difference between the affected side and normal side.

Neurovascular contact was defined as the absence of a cerebrospinal fluid signal gap between the nerve and the specific vessel on MRI (high-resolution T2W-3D-DRIVE sequence) [1]. Neurovascular contact was further classified into 3 types according to the Chavda system [20]: ① type I: the anterior inferior cerebellar artery (AICA) loop lies within the CPA but does not enter the IAC; ② type II: the AICA loop enters the IAC but does not extend more than 50% of the long axis of the IAC; and ③ type III: the AICA loop extends more than 50% into the IAC.

### Statistical analysis

Data were analyzed using SPSS v. 23.0 (Chicago, IL, USA), and a *p* value of < 0.05 (two-tailed) was considered statistically significant. Normally distributed continuous variables are reported as the mean ± standard deviation (SD), and non-normally distributed data are reported as the median and interquartile range (IQR). Continuous data were analyzed using Student’s *t*-test or the Mann–Whitney *U* test, and categorical data were analyzed using a 2 × 2 contingency table and Fisher’s exact test. Receiver operating characteristic (ROC) curves were calculated for ABR results to determine the most qualified variables, and the cutoff values were determined by maximizing the Youden index for sensitivity and specificity optimization to predict injury to the 8th cranial nerve in VP patients with neurovascular contact.

## Results

### Demographic characteristics

Thirteen patients with VP (8 [61.5%] males, 5 [38.5%] females) and 66 patients as controls (non-VP) were enrolled. In the VP group, twelve patients received oral oxcarbazepine, and one patient received carbamazepine because of nausea and vomiting with oxcarbazepine. All VP patients have received satisfactory pharmacological treatment efficacy thus far. Specifically, vertigo was controlled, and most typewriter tinnitus disappeared. However, in nine patients (69.2%), vertigo relapsed after discontinuation of the drug. The symptoms could be controlled again after restarting the medication at the original dose, and these patients adhered to drug therapy. The average follow-up time was 13.2 ± 5.8 months, ranging from 10 to 32 months. Detailed information on the VP patients is shown in Table 1.

**Table 1:** Democratic characteristics of patients with VP

No	Sex	Age (y)	Duration (y)	Lateral	Other symptoms	Neurovascular contact type	PTA	Caloric test		o-VEMP		c-VEMP		Pharmacological treatment scheme	Treatment outcomes	Follow up (months)
								CP (%)	DP (%)	Latency (n1, ms)	Amplitude (uA)	Latency (p1, ms)	Amplitude (uA)			
1	Male	55	1	Right	Typewriter tinnitus, hearing loss	Type I	31.3	10	21	9.7	4.5	13.4	94.6	Oxcarbazepine, 300 mg, bid, for 1 month	No vertigo attack has happened thus far and the tinnitus loudness decreased	11
2	Male	35	1	Right	Typewriter tinnitus, hearing loss	Type I	12.5	100	0	9.5	6.3	13.3	85.9	Oxcarbazepine, 300 mg, bid for 1 month	No vertigo attack has happened thus far; the tinnitus decreased	14
3	Male	58	7	Left	Typewriter tinnitus, hearing loss	Type I	75.0	54	3	11.8	2.1	Absent response	Absent response	Oxcarbazepine, 300 mg, bid, for 2 months; retaking consistently since relapse	The vertigo and tinnitus disappeared after 2-month therapy, but relapsed after another 2 months; no vertigo attack has happened since retaking the medication	11
4	Female	68	30	Left	Hearing loss	Type II	37.5	11	11	10.7	2.6	12.3	23.4	Oxcarbazepine, 300 mg, bid, for 1 month	No vertigo attack has happened thus far	11
5	Male	71	1	Right	Hearing loss	Type I	55.0	13	25	10.7	2.3	15.5	64.3	Oxcarbazepine, 300 mg, bid, for 3 months	No vertigo attack has happened thus far	11
6	Male	61	1.5	Left	Typewriter tinnitus, hearing loss	Type II	27.5	10	5	10.7	4.1	13.2	85.2	Oxcarbazepine, 300 mg, bid, consistently	The vertigo and tinnitus disappeared	11
7	Male	60	2	Left	Hearing loss	Type I	62.5	10	10	10.6	1.2	Absent response	Absent response	Oxcarbazepine, 300 mg, bid, consistently	Vertigo happened only once	12
8	Female	73	1	Right	Typewriter tinnitus, hearing loss	Type I	40.0	62	8	10	10.9	15.7	193.9	Oxcarbazepine, 300 mg, bid, for 1 month; retaking consistently since relapse	The vertigo and tinnitus disappeared after 1-month of therapy, but relapsed soon after 1-month withdrawal; no vertigo has happened since retaking the medication	11
9	Female	74	1	Right	Typewriter tinnitus, hearing loss	Type II	27.5	3	3	Missing	Missing	Missing	Missing	Carbamazepine, 200 mg, bid, for 1 month; retaking consistently since relapse	The vertigo and tinnitus disappeared after 1-month of therapy, but relapsed after 10 months; no vertigo attack has happened since restarting the medication	14
10	Male	32	0.2	Left	Typewriter tinnitus	Type I	6.3	13	13	9.1	7.0	13.2	121.7	Oxcarbazepine, 300 mg, bid, for 1 month; retaking for 2 months since relapse	The vertigo and tinnitus disappeared after 1-month of therapy, but relapsed soon after withdrawal; no vertigo attack has happened since retaking the medication	11
11	Male	33	0.1	Left	Typewriter tinnitus, hearing loss, tongue numbness	Type I	15.0	83	17	Absent response	Absent response	11.1	24.6	Oxcarbazepine, 300 mg, bid, consistently	The vertigo and tinnitus disappeared	12
12	Female	56	6	Left	Typewriter tinnitus, hemifacial spasm	Type III	26.3	Missing	Missing	Missing	Missing	Missing	Missing	Oxcarbazepine, 300 mg, bid, consistently	The vertigo and tinnitus disappeared	32
13	Female	50	0.2	Right	Typewriter tinnitus	Type I	26.3	67	29	Absent response	Absent Response	16.6	52.9	Oxcarbazepine, 300 mg, bid, consistently	The vertigo and tinnitus disappeared	10

*VP*, vestibular paroxysmia; *PTA*, pure tone audiometry; *CP*, canal paresis; *DP*, directional preponderance; *o-VEMP*, ocular vestibular evoked myogenic potential; *c-VEMP*, cervical vestibular evoked myogenic potential; *bid*, twice a day

The average age in the VP group was 55.8 ± 14.7 years, which was higher than that in the non-VP group (45.7 ± 11.4 years) (*p* = 0.016). A total of 76.9% of VP patients had typewriter tinnitus, but none of the patients in the control group experienced this type of tinnitus (*p* < 0.001). No obvious differences were found in vertigo type between the VP and non-VP groups (*p* = 0.732). Moreover, there were no significant differences in sex, duration, or laterality between the two groups (*p* > 0.05) (Table 2).

**Table 2:** Comparison between patients with VP and patients without VP

Valuables	Non-VP (*n* = 66)	VP (*n* = 13)	*p*
Gender (*n*, %)			
Male	23 (34.8)	8 (61.5)	0.072
Female	43 (65.2)	5 (38.5)	
Age (mean ± SD, y)	45.7 ± 11.4	55.8 ± 14.7	0.016*
Duration (mean ± SD, y)	5.1 ± 7.3	4.3 ± 8.4	0.304
Lateral (*n*, %)			
Left	27 (40.9)	7 (53.8)	0.389
Right	39 (59.1)	6 (46.2)	
Tinnitus (*n*, %)			
Typewriter tinnitus	0 (0.0)	10 (76.9)	< 0.001*
Pulsatile tinnitus	2 (3.0)	0 (0.0)	
Persistent tinnitus (low- or high- frequency)	21 (31.8)	0 (0.0)	
Vertigo (*n*, %)			
Acute vestibular syndrome	6 (9.1)	0 (0.0)	0.732
Episodic vestibular syndrome	47 (71.2)	13 (100.0)	
Chronic vestibular syndrome	2 (3.0)	0 (0.0)	
Neurovascular contact type (*n*, %)			
Type I	41 (62.1)	9 (69.2)	0.579
Type II	22 (33.3)	3 (23.1)	
Type III	3 (4.6)	1 (7.7)	
PTA (mean ± SD, dB HL)	33.3 ± 24.4	34.0 ± 20.0	0.605
Caloric test			
Missing (*n*, %)	15 (22.7)	1 (7.7)	
Canal paresis (mean ± SD, %)	31.9 ± 25.4	36.3 ± 34.5	0.681
Directional preponderance (mean ± SD, %)	14.8 ± 9.9	12.0 ± 9.1	0.373
o-VEMP			
Missing (*n*, %)	9 (13.6)	2 (15.4)	
Absent response (*n*, %)	4 (6.1)	2 (15.4)	
Response (*n*, %)	53 (80.3)	9 (69.2)	
Latency (mean ± SD, ms)	10.2 ± 0.9	7.1 ± 5.0	0.212
Amplitude (mean ± SD, uA)	3.3 ± 2.4	4.6 ± 3.1	0.210
c-VEMP			
Missing (*n*, %)	9 (13.6)	2 (15.4)	
Absent response (*n*, %)	8 (12.1)	2 (15.4)	
Response (*n*, %)	49 (74.2)	9 (69.2)	
Latency (mean ± SD, ms)	13.8 ± 2.1	13.8 ± 1.8	0.723
Amplitude (mean ± SD, uA)	64.9 ± 37.6	83.0 ± 52.7	0.316
ABR			
Affected side			
I wave latency (mean ± SD, ms)	1.7 ± 0.2	1.7 ± 0.1	0.530
Absent response (*n*, %)	6 (9.1)	2 (15.4)	
Response (*n*, %)	60 (90.9)	11 (84.6)	
III wave latency (mean ± SD, ms)	3.9 ± 0.2	4.3 ± 0.3	< 0.001*
Absent response (*n*, %)	3 (4.5)	0 (0.0)	
Response (*n*, %)	63 (95.5)	13 (100.0)	
V wave latency (mean ± SD, ms)	5.7 ± 0.2	6.1 ± 0.3	< 0.001*
Absent response (*n*, %)	2 (3.0)	0 (0.0)	
Response (*n*, %)	64 (97.0)	13 (100.0)	
IPL I–III (mean ± SD, ms)	2.1 ± 0.1	2.5 ± 0.3	< 0.001*
IPL III–V (mean ± SD, ms)	1.8 ± 0.2	1.8 ± 0.1	0.456
Normal side			
IPL I–III (mean ± SD, ms)	2.1 ± 0.1	2.2 ± 0.1	0.062
IPL III–V (mean ± SD, ms)	1.9 ± 0.2	1.9 ± 0.2	0.788

^*^p < 0.05

*VP*, vestibular paroxysmia; *PTA*, pure tone audiometry; *o-VEMP*, ocular vestibular evoked myogenic potential; *c-VEMP*, cervical vestibular evoked myogenic potential; *ABR*, auditory brainstem response; *IPL*, interpeak latency

### ABR results

Regarding the ABR results, there was no difference in the wave I latency of the affected side between the two groups (*p* = 0.530), but the wave III latency in the VP group was longer than that in the non-VP group (*p* < 0.001). Accordingly, the wave interval I–III in the VP group was longer than that in the non-VP group (*p* < 0.001). There was also a significant difference in wave V latency between the two groups (*p* < 0.001), but wave interval III–V showed no statistically significant difference (*p* = 0.456, see Table 2).

When comparing the differences between the affected and normal sides within the VP group, the wave III latency and IPL I–III of the affected side were longer than those of the normal side (*p* = 0.006; *p* = 0.001). No difference was observed in wave I and V latency or IPL III–V (*p* > 0.05, see Table 3).

**Table 3:** Comparison of ABR results between the normal side and affected side of the patients in the VP group (*n*=13)

Valuables (mean ± SD, ms)	Normal side	Affected side	*p*
I wave latency	1.8 ± 0.2	1.7 ± 0.1	0.165
III wave latency	4.0 ± 0.1	4.3 ± 0.3	0.006*
V wave latency	5.9 ± 0.2	6.1 ± 0.3	0.082
IPL I–III	2.2 ± 0.1	2.5 ± 0.3	0.001*
IPL III–V	1.9 ± 0.2	1.8 ± 0.1	0.236

^*^p < 0.05

*VP*, vestibular paroxysmia; *IPL*, interpeak latency

According to the criteria by Møller [22], a total of 84.6% of the VP patients and 4.5% of the non-VP controls had an IPL I–III ≥ 2.3 ms (*p* < 0.001). Furthermore, an IPL I–III difference between the affected side and the normal side ≥ 0.2 ms was more frequent in the VP group than in the non-VP group (*p* = 0.001). None of the VP patients had a contralateral IPL III–V ≥ 2.2 ms or IPL III–V difference ≥ 0.2 ms, with no differences compared with the control group (Table 4).

**Table 4:** Comparison of ABR results between the VP group and the non-VP group using Møller criteria

Variables	Non-VP (*n* = 66)	VP (*n* = 13)	*p*
IPL I–III of affected side (*n*, %)			
Absent response	6 (9.1)	2 (15.4)	< 0.001*
< 2.3 ms	57 (86.4)	0 (0.0)	
≥ 2.3 ms	3 (4.5)	11 (84.6)	
IPL III–V of normal side (*n*, %)			
Absent response	0 (0.0)	1 (7.7)	0.304
< 2.2 ms	65 (98.5)	12 (92.3)	
≥ 2.2 ms	1 (1.5)	0 (0.0)	
IPL I–III difference between the affected side and normal side (*n*, %)			
Absent response	6 (9.1)	3 (23.0)	0.001*
< 0.2 ms	56 (84.8)	5 (38.5)	
≥ 0.2 ms	4 (6.1)	5 (38.5)	
IPL III–V difference between the affected side and normal side (*n*, %)			
Absent response	2 (3.0)	1 (7.7)	0.498
< 0.2 ms	60 (90.9)	12 (92.3)	
≥ 0.2 ms	4 (6.1)	0 (0.0)	

^*^p < 0.05

*VP*, vestibular paroxysmia; *IPL*, interpeak latency

**Fig. 1:**
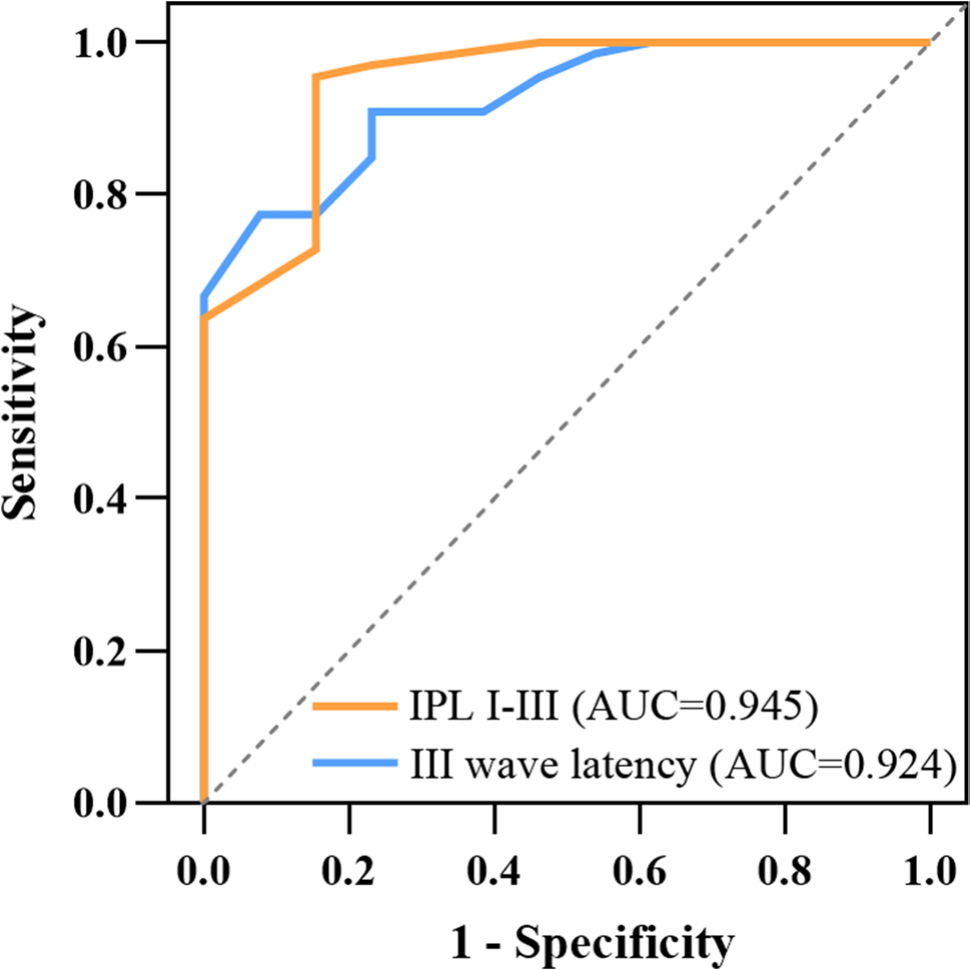
Receiver operating characteristic curves. Optimal points of IPL I–III and wave III latency were calculated according to the max Youden Index (Youden Index = Sensitivity + Specificity − 1)

After comparing the areas under the curve (AUCs) of wave latencies (affected side: I, III, and V) and wave intervals (affected side: I–III and III–V; normal side: III–V) by ROC analysis, we identified IPL I–III and wave III latency as the best predictive parameters for VP with neurovascular contact by MRI in our dataset (AUC > 0.9, see Table 5 and Supplemental Table 1). According to the ROC analyses, the optimal cutoff for IPL I–III was 2.3 ms (sensitivity 84.6%, specificity 95.5%), and wave III latency was 4.0 ms (sensitivity 92.3%, specificity 77.3%) (Fig. 1).

**Table 5:** Analysis of the receiver operating characteristic (ROC) curves

Variables	AUC	*p*
I wave latency of affected side	0.473	0.782
III wave latency of affected side	0.928	0.000*
V wave latency of affected side	0.853	0.000*
IPL I–III of affected side	0.993	0.000*
IPL III–V of affected side	0.398	0.306
IPL III–V of normal side	0.573	0.460

^*^p < 0.05

*IPL*, interpeak latency; *AUC*, area under the curve

### Other audiologic, vestibular, and imaging measurements

The average hearing threshold of the VP group was 34.0 ± 20.0 dB HL and that of the non-VP group was 33.3 ± 24.4 dB HL (*p* = 0.605). There were no significant differences in the canal paresis or directional preponderance of the caloric test (*p* = 0.681, *p* = 0.373). The latencies and amplitudes of o-VEMP and c-VEMP between the two groups showed no statistically significant differences (*p* > 0.05). Moreover, no difference in neurovascular contact type on MRI was observed between the VP and non-VP groups (*p* = 0.579) (Table 2).

## Discussion

VP is a rare episodic peripheral vestibular disorder that has a substantial impact on the quality of life of patients. Vascular compression of the cochleovestibular nerve is the widely accepted pathogenesis of VP. The symptoms are triggered by ephaptic transmission between demyelinated axons due to the pulsatile compression of the responsible vessel [19, 23]. A loop of the AICA seems to be the most relevant vessel (77.8–100%), followed by the posterior inferior cerebellar artery (PICA), the vertebral artery and veins [8, 10, 26]. Radiology is essential for a clear view of neurovascular contact, but the pressure of the vessels on nerves cannot be confirmed only by imaging*.* The present study was designed to analyze the value of electrophysiological tests in indicating pathogenic neurovascular contact in definite VP cases, with the goal of providing some objective references when decompression surgery is considered.

Previous articles reported some characteristics of ABR in VP patients, and the prolongation of IPL I–III was identified as a general and prevalent phenomenon [22, 24]. In the present study, we primarily found significant prolongation of IPL I–III and wave III and V latency in the VP group compared with the non-VP group. IPL III–V showed no statistically significant difference between the two groups (*p* = 0.456), indicating that the elongation of wave V latency in the VP group was the result of prolonged wave III. Additionally, according to the AUCs of these variables by ROC analysis, only IPL I–III and wave III latency were chosen for further assessment. Besides, we found that IPL I–III was the optimal parameter for diagnosing VP with satisfactory sensitivity and specificity (specificity = 95.5%; sensitivity = 84.6%), followed by wave III latency (specificity = 77.3%; sensitivity = 92.3%). As the absence of wave I was not rare in VP patients, we found that a prolonged wave III latency had a similar indicative value as a prolonged IPL I–III. The results suggested that neurovascular contact was more likely to be pathological in patients with VP-like clinical symptoms accompanied by either IPL I–III ≥ 2.3 ms or wave III latency ≥ 4.0 ms on the affected side.

More patients in the VP group (84.6%) had a value of IPL I–III ≥ 2.3 ms than patients in the non-VP group (4.5%) in this study. An absolute value of IPL I–III exceeding 2.3 ms was one of the Møller’s criteria for cochleovestibular compression syndrome in disabling positional vertigo and tinnitus [22], which coincides with the cutoff threshold determined in this study. The reasons for this similarity may be as follows: ① due to the rare incidence of VP, the sample size of this study was small, which may influence the values, and ② the high IPL I–III (≥ 2.3 ms) was not an indicator of VP exclusively but a possible indicator of a lesion or injury of the 8th cranial nerve. The scientific value of this study is twofold: ① the study focused on VP based on the new definition proposed in 2016, rather than a generic scope of diseases including vertigo and tinnitus, and ② the present results indicate that wave III latency ≥ 4.0 ms is an equally significant indicator of VP, useful when wave I is absent, which was not mentioned in Møller’s criteria. The contralateral IPL III–V may need to be longer to compensate for the abnormality of the affected side, as explained by De Ridder D. et al. [6]. However, no such differences in the IPL III–V of the normal side between the two groups were discovered, which needs further discussion.

There were no differences in the other acoustic and vestibular electrophysiological tests, such as PTA, caloric test, o-VEMP, and c-VEMP, between the VP and non-VP groups. In the literature, changes in these tests were controversial and did not show characteristic results in VP patients [5, 7, 10, 30]. Therefore, their potential to predict a neurovascular conflict in VP remains to be discussed. The present study found no difference in the type of vascular loop according to Chavda [20] between the VP group and the non-VP group (*p* = 0.579). Previous studies have demonstrated that there was no statistically significant link between the type of vascular loop and symptoms [17, 29]. As the type of vascular loop was not the major subject of this study, the correlation between vascular compression loci and VP has to be further evaluated with more nuanced measurement and typing on radiology.

The average age of VP patients was higher than that of non-VP patients. As reported previously, aging may cause vascular stiffening, leading to neurovascular conflict, possibly contributing to the onset of VP [12]. In addition, 76.9% of the patients had concomitant tinnitus, and all were of the typewriter kind. In contrast, none of the non-VP patients experienced typewriter tinnitus (*p* < 0.001). Our findings replicated the finding of a previous study showing that typewriter tinnitus is highly related to neurovascular compression of the 8th cranial nerve [15]. Mathiesen and Brantberg reported an important case with typewriter tinnitus and neurovascular conflict by MRI on the affected side, underwent microvascular decompression, and had long-term relief of the symptoms. This case suggested that typewriter tinnitus may be a useful indicator of neurovascular contact of the 8th cranial nerve and may be released by surgery [18]. Whether typewriter tinnitus can be used as an indicator for surgical treatment of VP remains to be explored. Most patients in this series of cases had to take medication consistently because vertigo recurred once drug discontinuance occurred at an interval time ranging from a few days to 10 months. It seemed that the symptoms easily relapsed after drug withdrawal in VP patients, so a relatively long-term follow-up was recommended for VP patients.

A limitation of this study was that as a preliminary study, the efficacy of ABR results for indicating neurovascular compression was not proven by surgery. Further studies on this issue are needed and planned in the future.

## Conclusion

The combination of prolonged IPL I–III or wave III latency of ABR and radiological findings strongly suggested that the MRI-verified vascular loop exerted symptomatic compression on the eighth cranial nerve in VP patients, which might guide the surgeon in decision-making regarding the indication for microvascular decompression in cases when medical therapy alone is not adequate. As the absence of wave I is not rare in VP patients, prolonged wave III latency is considered to serve as an equally significant indicator of VP as IPL I–III.

## Supplementary Information

Below is the link to the electronic supplementary material.

Supplementary file1 (DOCX 35 KB)

## Data Availability

The datasets generated during and/or analyzed during the current study are available from the corresponding author on reasonable request.
