# Supplementary material for: Application of ABR in pathogenic neurovascular compression of the 8th cranial nerve in vestibular paroxysmia
Source: Acta Neurochir (Wien). 2022 Mar 5;164(11):2953–62. doi: 10.1007/s00701-022-05157-2 (PMC9613544; doi:10.1007/s00701-022-05157-2)
Supplement: Supplementary file 1 — Supplementary file1 (DOCX 35 KB) [file 701_2022_5157_MOESM1_ESM.docx]

### **Supplemental Table 1**. Sensitivity and Specificity for I wave latency, III wave latency, V wave latency, IPL I-III and IPL III-V of affected side, and IPL III-V of normal side.

| **I wave latency of affected side (ms)** | | | **III wave latency of affected side (ms)** | | | **V wave latency of affected side (ms)** | | | **IPL I-III of affected side (ms)** | | | **IPL III-V of affected side (ms)** | | | **IPL III-V of normal side (ms)** | | |
| --- | --- | --- | --- | --- | --- | --- | --- | --- | --- | --- | --- | --- | --- | --- | --- | --- | --- |
| **Threshold** | **Sensitivity** | **Specificity** | **Threshold** | **Sensitivity** | **Specificity** | **Threshold** | **Sensitivity** | **Specificity** | **Threshold** | **Sensitivity** | **Specificity** | **Threshold** | **Sensitivity** | **Specificity** | **Threshold** | **Sensitivity** | **Specificity** |
| 0.4900 | 1.000 | 0.000 | 2.5200 | 1.000 | 0.000 | 4.3200 | 1.000 | 0.000 | 0.7000 | 1.000 | 0.000 | 0.5000 | 1.000 | 0.000 | .5800 | 1.000 | 0.000 |
| 1.5100 | 1.000 | 0.017 | 3.5250 | 1.000 | 0.017 | 5.3250 | 1.000 | 0.017 | 1.7600 | 1.000 | 0.017 | 1.5150 | 1.000 | 0.017 | 1.6000 | 1.000 | 0.017 |
| 1.5500 | 1.000 | 0.150 | 3.5500 | 1.000 | 0.033 | 5.3450 | 1.000 | 0.033 | 1.8250 | 1.000 | 0.033 | 1.5350 | 0.900 | 0.017 | 1.6600 | 1.000 | 0.067 |
| 1.5950 | 1.000 | 0.217 | 3.5900 | 1.000 | 0.067 | 5.3800 | 1.000 | 0.083 | 1.8300 | 1.000 | 0.050 | 1.5600 | 0.900 | 0.033 | 1.7050 | 1.000 | 0.083 |
| 1.6400 | 0.900 | 0.317 | 3.6350 | 1.000 | 0.117 | 5.4250 | 1.000 | 0.100 | 1.8550 | 1.000 | 0.067 | 1.5850 | 0.900 | 0.067 | 1.7100 | 0.900 | 0.100 |
| 1.6800 | 0.600 | 0.383 | 3.6800 | 1.000 | 0.167 | 5.4650 | 1.000 | 0.183 | 1.9400 | 1.000 | 0.083 | 1.6050 | 0.900 | 0.100 | 1.7250 | 0.900 | 0.133 |
| 1.7200 | 0.300 | 0.533 | 3.7200 | 1.000 | 0.267 | 5.4850 | 1.000 | 0.200 | 2.0000 | 1.000 | 0.100 | 1.6400 | 0.900 | 0.133 | 1.7450 | 0.900 | 0.167 |
| 1.7600 | 0.100 | 0.683 | 3.7600 | 1.000 | 0.333 | 5.5100 | 0.900 | 0.250 | 2.0200 | 1.000 | 0.167 | 1.6650 | 0.900 | 0.150 | 1.7500 | 0.800 | 0.250 |
| 1.8000 | 0.100 | 0.767 | 3.8000 | 1.000 | 0.383 | 5.5500 | 0.900 | 0.333 | 2.0400 | 1.000 | 0.167 | 1.6850 | 0.900 | 0.167 | 1.7650 | 0.800 | 0.317 |
| 1.8450 | 0.100 | 0.800 | 3.8250 | 1.000 | 0.417 | 5.5900 | 0.900 | 0.367 | 2.0600 | 1.000 | 0.317 | 1.7050 | 0.900 | 0.183 | 1.7850 | 0.700 | 0.317 |
| 1.8900 | 0.000 | 0.833 | 3.8450 | 1.000 | 0.433 | 5.6300 | 0.900 | 0.383 | 2.1000 | 1.000 | 0.450 | 1.7300 | 0.700 | 0.200 | 1.7900 | 0.600 | 0.383 |
| 1.9300 | 0.000 | 0.850 | 3.8850 | 1.000 | 0.583 | 5.6750 | 0.900 | 0.467 | 2.1250 | 1.000 | 0.483 | 1.7500 | 0.700 | 0.233 | 1.7950 | 0.600 | 0.450 |
| 1.9700 | 0.000 | 0.867 | 3.9300 | 1.000 | 0.683 | 5.7200 | 0.900 | 0.517 | 2.1300 | 1.000 | 0.483 | 1.7700 | 0.700 | 0.283 | 1.8150 | 0.600 | 0.467 |
| 2.0100 | 0.000 | 0.917 | 3.9550 | 0.900 | 0.800 | 5.7450 | 0.900 | 0.633 | 2.1450 | 1.000 | 0.550 | 1.7950 | 0.500 | 0.333 | 1.8350 | 0.600 | 0.500 |
| 2.0500 | 0.000 | 0.967 | 3.9750 | 0.800 | 0.800 | 5.7650 | 0.800 | 0.633 | 2.1650 | 1.000 | 0.633 | 1.8050 | 0.400 | 0.333 | 1.8550 | 0.600 | 0.533 |
| 2.1550 | 0.000 | 0.983 | 4.0100 | 0.700 | 0.883 | 5.8000 | 0.800 | 0.717 | 2.1900 | 1.000 | 0.700 | 1.8150 | 0.400 | 0.350 | 1.8700 | 0.500 | 0.550 |
| 3.2400 | 0.000 | 1.000 | 4.0500 | 0.700 | 0.900 | 5.8400 | 0.800 | 0.817 | 2.2300 | 1.000 | 0.817 | 1.8250 | 0.400 | 0.367 | 1.8750 | 0.500 | 0.567 |
|  |  |  | 4.0900 | 0.700 | 0.950 | 5.8800 | 0.800 | 0.833 | 2.2700 | 1.000 | 0.917 | 1.8300 | 0.300 | 0.517 | 1.8950 | 0.400 | 0.567 |
|  |  |  | 4.1350 | 0.600 | 0.950 | 5.9250 | 0.700 | 0.867 | 2.2950 | 1.000 | 0.950 | 1.8350 | 0.300 | 0.533 | 1.9150 | 0.400 | 0.700 |
|  |  |  | 4.1800 | 0.500 | 0.950 | 5.9700 | 0.700 | 0.917 | 2.3150 | 0.900 | 0.967 | 1.8550 | 0.300 | 0.550 | 1.9350 | 0.400 | 0.750 |
|  |  |  | 4.2200 | 0.500 | 0.983 | 6.0100 | 0.500 | 0.933 | 2.3700 | 0.700 | 1.000 | 1.8700 | 0.300 | 0.600 | 1.9550 | 0.400 | 0.767 |
|  |  |  | 4.2600 | 0.400 | 1.000 | 6.0500 | 0.500 | 0.983 | 2.4150 | 0.600 | 1.000 | 1.8750 | 0.300 | 0.617 | 1.9600 | 0.400 | 0.800 |
|  |  |  | 4.3000 | 0.300 | 1.000 | 6.1100 | 0.400 | 0.983 | 2.4350 | 0.500 | 1.000 | 1.8800 | 0.200 | 0.633 | 1.9750 | 0.400 | 0.867 |
|  |  |  | 4.4050 | 0.200 | 1.000 | 6.1750 | 0.300 | 1.000 | 2.4950 | 0.400 | 1.000 | 1.8950 | 0.200 | 0.667 | 1.9950 | 0.300 | 0.900 |
|  |  |  | 4.6750 | 0.100 | 1.000 | 6.3850 | 0.100 | 1.000 | 2.5800 | 0.300 | 1.000 | 1.9100 | 0.100 | 0.717 | 2.0200 | 0.300 | 0.917 |
|  |  |  | 5.8600 | 0.000 | 1.000 | 7.5700 | 0.000 | 1.000 | 2.6850 | 0.200 | 1.000 | 1.9150 | 0.100 | 0.733 | 2.0600 | 0.200 | 0.917 |
|  |  |  |  |  |  |  |  |  | 2.9550 | 0.100 | 1.000 | 1.9400 | 0.100 | 0.783 | 2.0850 | 0.100 | 0.917 |
|  |  |  |  |  |  |  |  |  | 4.1600 | 0.000 | 1.000 | 1.9600 | 0.100 | 0.800 | 2.1050 | 0.100 | 0.933 |
|  |  |  |  |  |  |  |  |  |  |  |  | 1.9750 | 0.100 | 0.833 | 2.1200 | 0.100 | 0.933 |
|  |  |  |  |  |  |  |  |  |  |  |  | 1.9950 | 0.100 | 0.850 | 2.1450 | 0.000 | 0.967 |
|  |  |  |  |  |  |  |  |  |  |  |  | 2.0000 | 0.100 | 0.850 | 2.4350 | 0.000 | 0.983 |
|  |  |  |  |  |  |  |  |  |  |  |  | 2.0200 | 0.000 | 0.917 | 3.7000 | 0.000 | 1.000 |
|  |  |  |  |  |  |  |  |  |  |  |  | 2.0800 | 0.000 | 0.950 |  |  |  |
|  |  |  |  |  |  |  |  |  |  |  |  | 2.1250 | 0.000 | 0.967 |  |  |  |
|  |  |  |  |  |  |  |  |  |  |  |  | 2.1650 | 0.000 | 0.983 |  |  |  |
|  |  |  |  |  |  |  |  |  |  |  |  | 3.2000 | 0.000 | 1.000 |  |  |  |
